# S14-3 Finnish On the Move Programme Family: Finnish Schools on the Move

**DOI:** 10.1093/eurpub/ckad133.069

**Published:** 2023-09-11

**Authors:** Päivi Aalto-Nevalainen

**Affiliations:** Ministry of Education and Culture, Finland

## Abstract

**Purpose:**

Finnish On the Move Programme promotes a physically active lifestyle throughout the lifespan for different demographic groups. It is financed by the Ministry of Education and Culture. One of the programmes of this entity is Finnish Schools on the Move, which is a research-based programme for promoting physical activity in schools.

**Project description:**

Schools on the Move programme has been developed in Finland since 2010 and has grown from a pilot of 45 schools into a programme that covers more than 90 per cent of Finnish schools in basic education.

In ten years, we have managed to increase the awareness of the importance of physical activity in Finland. More than 90 per cent of all comprehensive schools in Finland are Schools on the Move. As a result, the school culture has become more physically active: a positive change has been observed in the organisation of activities and school premises, movement has been added to lessons and more attention has been paid to less active pupils. Based on national level physical activity surveys the proportion of 11-15-years olds meeting the physical activity recommendation has slightly increased from 18% to 29% in girls and from 30% to 35% for boys between 2010 and 2018. (Finland’s Report Card 2022. Physical Activity for Children and Youth).

The Schools on the Move programme has been part of the implementation of the government programme in Finland since 2010. Between 2015 and 2019 it was one of the Government’s key projects in knowledge and education.

**Conclusions:**

The objective of the programme is to increase physical activity among school-age children by making the operating culture at schools more active in various ways. Linking research and monitoring with the implementation of the programme, the strong bottom-up ideology of the school-oriented approach and the extensive cooperation networks, as well as strong commitment from the government and state funding, have been central success areas in the implementation of the programme.

